# Modeling the differential phenotypes of spinal muscular atrophy with high-yield generation of motor neurons from human induced pluripotent stem cells

**DOI:** 10.18632/oncotarget.14925

**Published:** 2017-01-31

**Authors:** Xiang Lin, Jin-Jing Li, Wen-Jing Qian, Qi-Jie Zhang, Zhong-Feng Wang, Ying-Qian Lu, En-Lin Dong, Jin He, Ning Wang, Li-Xiang Ma, Wan-Jin Chen

**Affiliations:** ^1^ Department of Neurology and Institute of Neurology, First Affiliated Hospital, Fujian Medical University, Fuzhou 350005, China; ^2^ Institutes of Brain Science, Institute of Neurobiology, State Key Laboratory of Medical Neurobiology, Collaborative Innovation Center for Brain Science, Fudan University, Shanghai 200032, China; ^3^ Department of Anatomy, Histology & Embryology, Shanghai Medical College, Fudan University, Shanghai 200032, China; ^4^ Fujian Key Laboratory of Molecular Neurology, Fujian Medical University, Fuzhou 350005, China

**Keywords:** spinal muscular atrophy (SMA), induced pluripotent stem cells (iPSCs) derived enriched motor neurons (MNs), survival motor neuron (SMN) protein, neurite outgrowth, neuronal activity

## Abstract

Spinal muscular atrophy (SMA) is a devastating motor neuron disease caused by mutations of the survival motor neuron 1 (*SMN1*) gene. *SMN2*, a paralogous gene to *SMN1*, can partially compensate for the loss of *SMN1*. On the basis of age at onset, highest motor function and *SMN2* copy numbers, childhood-onset SMA can be divided into three types (SMA I-III). An inverse correlation was observed between *SMN2* copies and the differential phenotypes of SMA. Interestingly, this correlation is not always absolute. Using SMA induced pluripotent stem cells (iPSCs), we found that the SMN was significantly decreased in both SMA III and SMA I iPSCs derived postmitotic motor neurons (pMNs) and γ-aminobutyric acid (GABA) neurons. Moreover, the significant differences of SMN expression level between SMA III (3 copies of *SMN2*) and SMA I (2 copies of *SMN2*) were observed only in pMNs culture, but not in GABA neurons or iPSCs. From these findings, we further discovered that the neurite outgrowth was suppressed in both SMA III and SMA I derived MNs. Meanwhile, the significant difference of neurite outgrowth between SMA III and SMA I group was also found in long-term cultures. However, significant hyperexcitability was showed only in SMA I derived mature MNs, but not in SMA III group. Above all, we propose that SMN protein is a major factor of phenotypic modifier. Our data may provide a new insight into recognition for differential phenotypes of SMA disease.

## INTRODUCTION

Spinal muscular atrophy (SMA) is a devastating disorder associated with selective degeneration of spinal motor neurons (MNs), resulting in progressive muscle atrophy, generalized weakness and often death [1]. It is one of the leading known genetic cause of infant mortality, affecting 1 in 6000 to 10 000 infants [2]. According to the age of onset, highest motor function achieved and survival motor neuron 2 (*SMN2*) gene copy numbers, childhood-onset SMA is classified into three types (SMA I-III) [3]. Since there are no effective treatment options, the severe type I patients (SMA I) usually die by the age of two.

SMA is caused by mutations in the survival motor neuron 1 (*SMN1*) gene, resulting in a deficiency of the ubiquitously expressed SMN protein [2–4]. Human SMN has two genes, *SMN1* and *SMN2*, but most animals have only *Smn1* gene. *SMN1* primarily generates full length of functional protein (SMN-FL). *SMN2* mostly (80~90%) produces the truncated, rapidly degraded protein lacking exon 7 (*SMN-Δ7*), and generates only 10% of SMN-FL [5]. Thus recent advances in cell reprogramming have enabled to establish a more appropriate human derived model for SMA disease than animal models [6–9]. Induced pluripotent stem cells (iPSCs) from SMA patients have been generated to study the motor neuron phenotypes in cell cultures [10–15]. However, these researches have only generated SMA I iPSC lines using fibroblasts from invasive biopsy, and have mainly focused on the phenotypes of mature motor neurons (mMNs). Meanwhile, the MN differentiation protocols they employed were time-consuming and low-efficient, which also limit SMA disease modeling studies.

SMN protein is involved in the biogenesis of small nuclear ribonucleoproteins (snRNPs) and mRNA splicing [16]. *SMN2* copy numbers are the most important known phenotypic modifier of SMA, since a higher number of *SMN2* copies correlates with higher production of functional SMN-FL protein [13, 17, 18]. Whereas this correlation is not always absolute in the phenotypic severity of SMA disease [19]. To elucidate the reason for differential phenotypic severity of SMA, we herein evaluated the relationship between SMN protein level and neurite outgrowth or neuronal activity, using the high-yield generation of postmitotic motor neurons (pMNs) and mMNs from SMA III and SMA I patients derived iPSCs.

## RESULTS

### Generation of patient-specific SMA III and SMA I iPSCs

**Figure 1:**
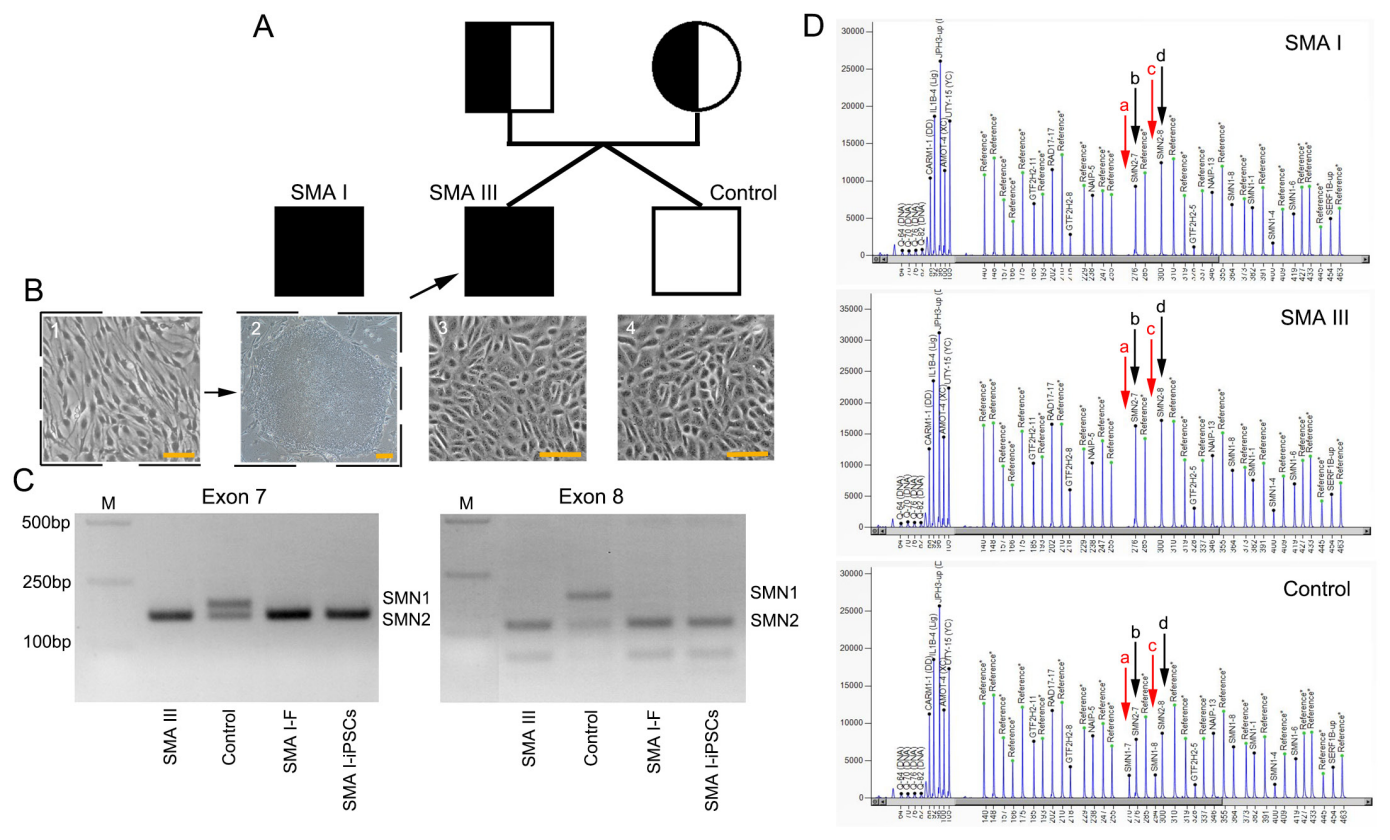
SMN gene mutation screening of cell lines from the three individuals **A**. A schematic diagram of the pedigree for iPS cell generation. In addition, iPS cells from a SMA I patient outside of the pedigree were used in the current study. **B**. Cell lines from the three individuals: iPSCs reprogrammed from fibroblasts (1-2, the SMA I patient), and primary culture of urine cell (3-4, the SMA III patient and healthy control). Scale bar, 100 μm. **C**. Detection of homozygous deletion of *SMN1* gene (exon 7 and exon 8) by PCR-RFLP. **D**. MLPA results, red arrows- “a” and “c” indicate *SMN1* gene (exon 7 and exon 8), black arrows- “b” and “d” indicate *SMN2* gene (exon 7 and exon 8).

Urine cells were obtained from a mild SMA patient and his unaffected male sibling (Figure 1A, 1B). The affected twin is 20 years old, with onset at approximately 20 months old. History of pregnancy and delivery was normal. Currently, he cannot walk without assistance. Physical examination showed symmetrical reduction in muscle strength in the neck and limbs and the disappearance of tendon reflexes. Further investigation via electromyography revealed extensive nerve damage. Mutation screening with genomic DNA from the urine cells by polymerase chain reaction-restriction fragment length polymorphism (PCR-RFLP) and multiplex ligation-dependent probe amplification (MLPA) showed homozygous deletion of *SMN1* and three copies of *SMN2*, respectively (Figure 1C, 1D). Additionally, the unaffected twin harbored two copies of *SMN1* and *SMN2* (Figure 1D). Eventually, the affected individual was diagnosed with mild type III SMA (SMA III) according to the latest diagnostic criteria [20].

**Figure 2:**
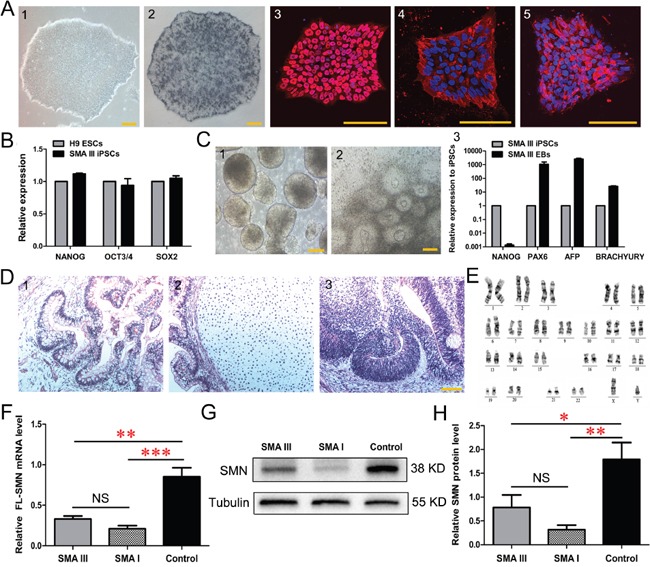
Characterization of urine derived-iPSC colonies (SMA III iPSCs) **A**. Alkaline phosphatase (AP) staining and immunofluorescence of selected iPSC lines (1) show the expression of the human ESC-specific markers: AP (2), NANOG (3), SSEA4 (4) and TRA-1-60 (5). **B**. The endogenous expression of the pluripotency genes (NANOG, OCT3/4 and SOX2) was quantified by qRT-PCR. Gene expression was normalized to the H9 ESCs, which was arbitrarily set to 1 (n=3). **C**. Phase contrast photographs show the formation of EBs (1) on Day 8 and rosettes (2) on Day 16 from iPSCs, and qPCR detected the genes PAX6 (ectoderm), AFP (endoderm) and BRACHYURY (mesoderm) reflecting the 3-germ layer differentiation of EBs (3) (n=3). **D**. The sections of teratomas were stained with hematoxylin-eosin: endoderm (1), mesoderm (2) and ectoderm (3). **E**. The karyotypes of selected iPSC colonies are normal. **F-H**. Quantitative PCR **(F)** and western blotting **(G, H)** analysis revealed a significantly decreased expression of SMN in SMA III and SMA I iPSC lines compared to the control iPSCs (n=5, one way ANOVA, *P < 0.05, **P < 0.01, ***P < 0.001). All data presented as the mean ± SEM. Scale bar, 100 μm.

Urine cell lines from the dizygotic twins were then reprogrammed to iPSCs using lentiviral constructs encoding *OCT4*, *SOX2*, *KLF4* and *cMYC* (Supplementary Figure 1A). The resulting cells displayed sequential morphological changes during the reprogramming process. These human embryonic stem (ES)-like cells could be passaged and expanded on Matrigel with defined boundaries (Figure 2A and Supplementary Figure 1B). The isolated clonies were named SMA III iPSCs and control iPSCs, respectively. All iPSC lines evaluated in these studies expressed pluripotent markers, including alkaline phosphatase (AP), NANOG, SSEA4 and TRA-1-60 (Figure 2A and Supplementary Figure 1B). These clones also expressed endogenous pluripotent genes (Figure 2B and Supplementary Figure 1C), maintained normal karyotypes (Figure 2E and Supplementary Figure 1F), and they could form 3 germ layers *in vitro* (Figure 2C and Supplementary Figure 1D) and *in vivo* (Figure 2D and Supplementary Figure 1E). Furthermore, the results of qPCR and western blotting showed that SMN mRNA and protein were significantly decreased in SMA III iPSC lines compared to the control iPSCs (Figure 2F, 2G, 2H), indicating that the mutant *SMN1* gene is maintained during reprogramming. DNA fingerprinting also confirmed their origin from parental urine cells without contamination (Supplementary Table 1). Thus, urine-derived iPSC lines from the two individuals have been efficiently generated.

Additionally, the severe SMA I iPSC line was from Coriell Cell Repositories (Figure 1A, 1B). Briefly, the cell line was derived from SMA type I patient's fibroblasts by reprogramming with lentiviral constructs encoding OCT4, SOX2, NANOG and LIN28 [10]. Molecular analysis of *SMN* gene showed homozygous deletion of *SMN1* and two copies of *SMN2*, respectively (Figure 1C, 1D).

### Induce SMA III and SMA I iPSCs to rapidly and efficiently differentiate into motor neurons

**Figure 3:**
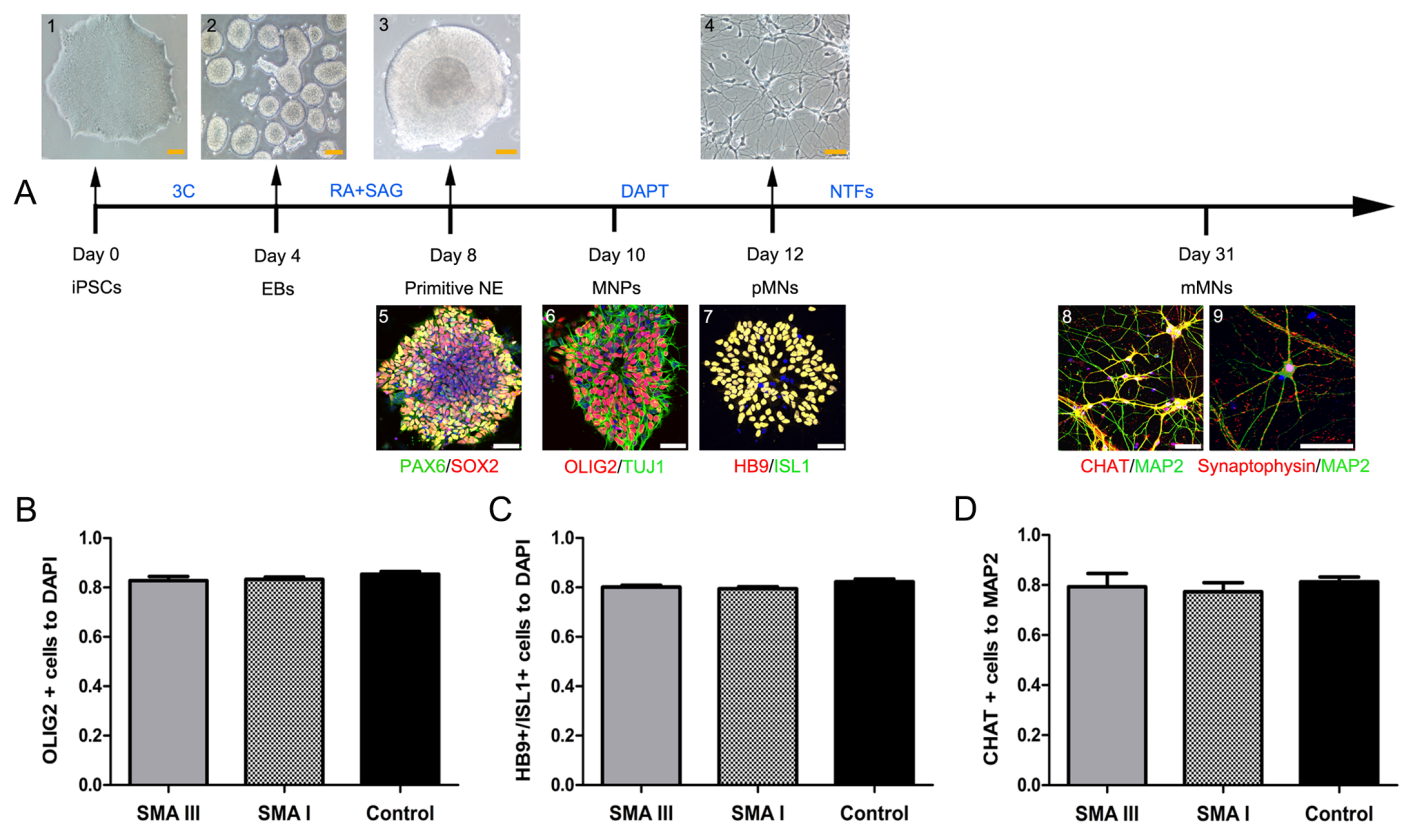
Stepwise differentiation of motor neurons **A**. Schematic diagram of motoneuron differentiation (SMA III iPSCs). Human PSCs (1) were induced to neuroectodermal EBs (2) in the presence of three small molecular compounds (3C) for 4 days, then were patterned to PAX6^+^/SOX2^+^ primitive neuroepithelia (NE, 3 and 4) and OLIG2^+^/TUJ1^+^ motor neuron progenitors (MNPs, 5) for 4-6 days with retinoic acid (RA) and SAG. The MNPs were then plated onto laminin substrate to generate HB9^+^/ISL1^+^ postmitotic MNs (6, 7) with DAPT for 3 days and to generate CHAT^+^/Synaptophysin^+^/MAP2^+^ mature mMNs (8, 9) with neurotrophic factors (NTFs) for approximately three weeks. **B-D**. Quantification of the percentage of OLIG2^+^ MNPs on Day 10 (B), HB9^+^/ISL1^+^ pMNs on Day 12 (C), and ChAT^+^ mMNs on Day 31 (D) showed no significant differences in differentiation efficiency (n=3, one way ANOVA, P > 0.5) among SMA III, SMAI and Control cultures. All data are represented as the mean ± SEM. Scale bar, 50 μm.

All of SMA III, SMA I and control iPSC lines were differentiated into spinal motor neurons using a modified differentiation protocol (Figure 3A). This method takes advantage of developmental principles to generate spinal MNs rapidly and efficiently. Human iPSCs were induced to a neural lineage by generating embryoid bodies (EBs), and then were cultured in a neural medium supplemented with compounds. To efficiently generate caudalized and ventralized neural precursors, RA and SAG were added to the neural differentiation medium on Day 4. After another 4 days of treatment, the PAX6^+^/SOX2^+^ primitive neuroepithelium (NE) formed with large, bright and tight morphology changes. As reported earlier, OLIG2^+^/TUJ1^+^spinal motor neuron progenitors (MNPs) were efficiently generated on Day 10 after differentiation. After a NOTCH inhibitor-DAPT treatment, a majority of cells already co-expressed pMN markers HB9 and ISL1 on Day 12. These motor neurons then matured *in vitro*, as indicated by immunostaining with mMN markers choline acetyltransferase (CHAT), MAP2 and Synaptophysin on Day 31.

To test whether the absence of the *SMN1* gene would inhibit the specification of spinal MNs, we further examined the proportions of OLIG2^+^ cells, HB9^+^/ISL1^+^ cells and CHAT^+^ cells. At these time points, there were no significant differences among all three group cultures in the number (~83%) of MNPs (Figure 3B and Supplementary Figure 2A), in the number (~80%) of pMNs (Figure 3C and Supplementary Figure 2B) or in the number (~79%) of mMNs (Figure 3D and Supplementary Figure 2C), respectively.

Taken together, the above results show that the absence of the *SMN1* gene does not alter the temporal differentiation and maturation of spinal MNs, and treatment with DAPT substantially accelerates and promotes pMN generation.

### Immunoblot analysis on SMN from SMA III and SMA I postmitotic MNs and GABA neurons

**Figure 4:**
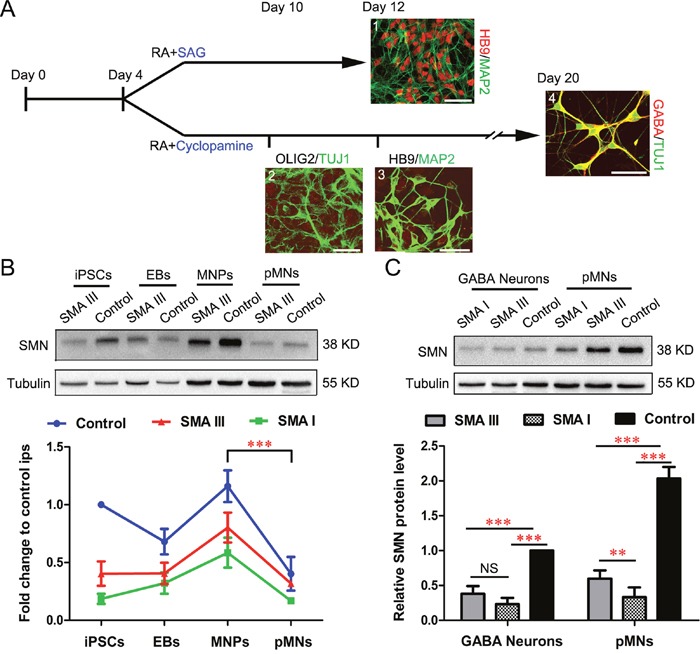
Enriched postmitotic MNs and GABA neurons for SMA modeling **A**. Schematics for postmitotic MNs (1) and postmitotic GABA neurons (4) differentiation (SMA III iPSCs). Immunofluorescent images of HB9^+^/MAP2^+^ pMNs (1) on Day 12, non-MNs for OLIG2^−^/TUJ1^+^ (2) on Day 10, HB9^−^/MAP2^+^ (3) on Day 12 and GABA^+^/TUJ1^+^ (4) on Day 20, respectively. Scale bar, 50 μm. **B**. Expression of SMN protein at different stages during differentiation of pMNs from SMA III, SMA I and Control iPSCs was examined by western blot (n=3). **C**. SMN protein levels were analyzed by western blot at the postmitotic MNs and GABA neurons and samples grouped as SMA III, SMA I or Control (n=5). All the groups were collected and sampled under the same conditions. All data are presented as the mean ± SEM. **P < 0.01, ***P < 0.001; one-way ANOVA was used for the data analysis.

After the enriched iPSC-derived pMNs were rapidly generated, we next examined the phenotypes of pMN culture in different types of SMA. To mimic selective vulnerability of motoneurons, pMNs and GABA neurons from the same iPSCs were generated by SAG and cyclopamine to activate or block SHH signaling, respectively (Figure 4A) [21]. Under the treatment of SAG, the cells expressed specific pMNs markers HB9 and MAP2 on Day 12. With the intervention of cyclopamine for 6 days, the induced neural precursors were void of OLIG2 expression on Day 10, and the postmitotic neurons were void of HB9 expression on Day12, but with GABA expression on Day 20.

To compare SMN protein expression levels among SMA III, SMA I and control groups during the iPSC derived MN differentiation, four different culture stages were collected, including iPSCs (Day 0), EBs (Day 4), MNPs (Day 10), and pMNs (Day 12). The results showed that SMN protein expressed highest in the MNP culture stage and then decreased significantly along the pMN differentiation, which existed in all three groups (Figure 4B). To further compare the expression levels of SMN protein between different neuronal types, all three groups derived postmitotic GABA neurons were also collected. Western blot analyses demonstrated a significant increase in the expression of SMN protein in pMNs compared with GABA neurons (Figure 4C). Meanwhile, we also found that the expression levels of SMN protein in both neuronal types derived from SMA groups were lower than control. This is consistent with the fact that various cell types were affected in SMA disease. Notably, the significant difference of SMN protein expression level between SMA III and SMA I was found in pMN culture, but not in GABA neurons or iPSCs (Figure 2G, 2H).

### Neurite outgrowth analysis on MN-enriched clusters from SMA III and SMA I iPSCs

**Figure 5:**
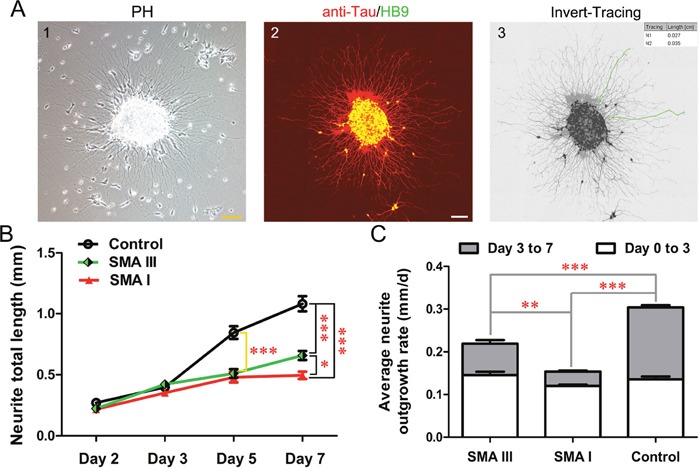
Neurite outgrowth analysis on iPSC derived pMN **A**. The morphology of neurites from attached neurosphere of SMA III on Day 2 after attachment (1) and method employed to perform neurite measurements (2, 3). Scale bar, 100 μm. **B**. Neurite total length were calculated from Day 2 to Day 7 after attachment of neurospheres (n=50, one-way ANOVA, *P < 0.05, ***P < 0.001). **C**. Average neurite outgrowth rate were compared at two intervals (Day 0 to Day 3 and Day 3 to Day 7 after attachment) among SMA III, SMA I and control groups (one -way ANOVA, **P < 0.01, ***P < 0.001). All data are presented as the mean ± SEM.

The differentiation of iPSCs into pMNs is characterized by morphological change in the radial projection of neurites from attached neurospheres. To investigate the specific differences among different types of SMA and control groups, the average neurite outgrowth rate and the total length of neurite for each cluster were analyzed from Day 2 to Day 7 after attachment of neurospheres on Day 9. These clusters were fixed and subjected to anti-Tau and HB9 co-immunostaining to label the specific MN neurite outgrowth, then were calculated by Image J (Figure 5A).

The results showed that control clones harbored trend with higher values in neurite total length than both SMA clones (Figure 5B). The differences reached statistical significance on Day 5 after attachment. Interestingly, neurites projected from SMA III clusters were much longer than that from SMA I clusters on Day 7. Furthermore, the average neurite outgrowth rate shared the same tendency as well. The significant difference appeared at the interval between Day 3 to Day 7 after attachment among all three groups (Figure 5C).

### Electrophysiological recording of SMA III and SMA I mature MNs

**Figure 6:**
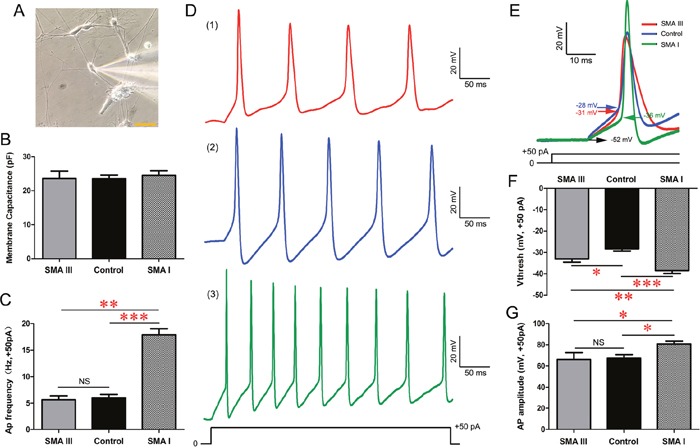
Excitability analysis of iPSC derived mature MNs by electrophysiology in current-clamp mode **A**. A representative image of electrophysiological recording on individual SMA III mMNs. Scale bar, 50 μm. **B**. Recording of average membrane capacitance for SMA III, SMA I and control groups. **C**. Statistical analysis of AP frequency in all three groups. **D**. Representative traces of induced APs at 50 pA current injections for SMA III (red, 1), SMA I (green, 3) and control (blue, 2) groups. **E**. Representative individual AP induced by + 50 pA injection for each group. Arrows indicate voltage threshold of AP. **F-G**. Statistical analysis of AP threshold **(F)** and AP amplitude **(G)** in all threee groups. n=18 neurons for each individual. All data shown represent mean ± SEM. *P < 0.05, **P < 0.01, ***P < 0.001; one-way ANOVA was used for the data analysis.

We next investigated whether the above differences were correlated with the electrophysiological properties of SMA III, SMA I and control iPSCs-derived mature MNs. After approximately 5 weeks of total differentiation, multipolar neurons with large cell bodies (Figure 6A), as indicated by cell capacitances of ~20 pF, were recorded (18 neurons/group) [22]. Whole cell patch-clamp recordings showed that there were no significant differences in membrane capacitance among these iPSCs-derived mMNs (Figure 6B).

In current-clamp mode, induced SMA I mMNs exhibited higher action potential (AP) frequency in response to a +50pA current injection than SMA III and control culture (Figure 6C, 6D). Detailed analysis of the kinetics of individual AP triggered by the +50pA current injection revealed that the AP threshold (arrows in Figure 6E) in both SMA III and SMA I mMNs were significantly reduced compared to that of control mMNs (Figure 6F). However, only the SMA I mMNs showed significant difference in AP amplitude to control mMNs (Figure 6G). Therefore, we further measured the Na^+^ channel activities that are known to play a crucial role in the activation of AP firing.

**Figure 7:**
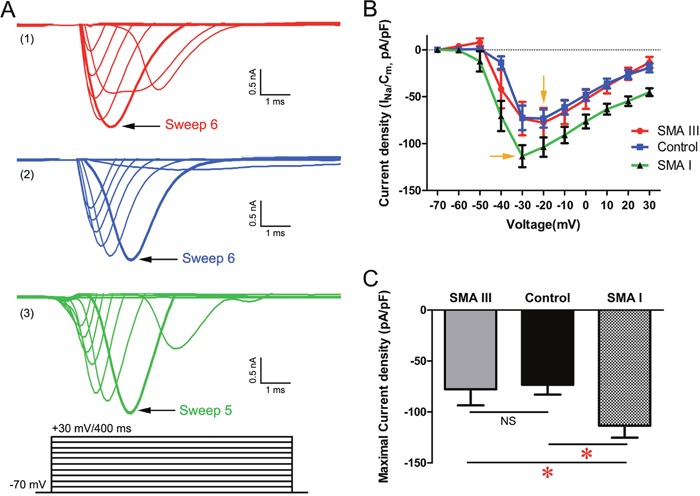
Excitability analysis of iPSC derived mature MNs by electrophysiology in voltage-clamp mode **A**. Sodium currents (I_Na_) were reliably elicited from a holding potential of -70 mV by 10 mV increments to +30 mV in 400 ms duration in SMA III (red, 1), SMA I (green, 3) and control (blue, 2) groups. **B**. Current voltage relations for the Na^+^ current density evoked between −70 mV and +30 mV in all three groups. **C**. Summary of the maximal Na^+^ current density for each group (one-way ANOVA, *P < 0.05). n=18 neurons for each individual. All data shown represent mean ± SEM.

In voltage-clamp mode, all the recorded mMNs displayed fast-activating, fast-inactivating inward Na^+^ currents (I_Na_) and large outward K^+^ currents (I_k_) (Figure 7A and Supplementary Figure 3A, 3B, 3C). The I_Na_ could be effectively blocked by the application of tetrodotoxin (TTX). To exclude the effect of heterogeneous membrane capacitance, we normalized the peak I_Na_ to its corresponding membrane capacitance as current density and then plotted with the depolarizing voltage potentials (Figure 7B). The results showed that the minimal voltage required to trigger maximal current density was -30 mV for SMA I mMNs compared to -20 mV for SMA III and control mMNs. Meanwhile, SMA I mMNs displayed significantly enhanced Na^+^ currents density compared to SMA III and control mMNs (Figure 7C).

In sum, our data demonstrate that both SMA iPSC-derived pMNs have the capacity to differentiate into functionally mature MNs with defined electrophysiological characteristics. Hyperexcitability of neuronal activity has been found only in SMA I mMNs, but not in SMA III mMNs.

## DISCUSSION

Human pluripotent stem cells, including embryonic stem cells (ESCs) and induced pluripotent stem cells (iPSCs), offer a new system model for dissecting the disease phenotypes [7, 8, 23]. To date, human iPSCs have been generated from various somatic cell types, such as skin fibroblasts, urine cells, keratinocytes, adipose stem cells, etc [24]. Compared with fibroblasts from skin biopsy, the un-invasive urine cells as reprogramming parental cell is much more acceptable. It has previously been reported that human iPSCs derived from different cellular origins (fibroblasts versus cord blood) showed indistinguishable pluripotency, but haboured biased tendency for lineage-specific differentiation [25]. However, the present study revealed that there were no significant differences in the specification and maturation of motor neurons between fibroblasts and urine cells derived SMA iPSCs. We speculate that it may be related to their same origin from ectoderm.

SMA, a motoneuron developmental disorder, is caused by decrease in the amount of SMN protein [26, 27]. Intriguingly, we found the SMN reduced significantly along pMN differentiation, but expressed much higher in pMNs than GABA neurons. Moreover, the significant differences of SMN concentration between SMA III (3 copies of SMN2) and SMA I (2 copies of SMN2) were observed only in pMN culture, but not in GABA neurons or iPSCs. *Ahmad* et al. raised that the differential expression of SMN has been related to the disparate transcription initiation sites (at the positions -163 and -246) in different cell types [28]. However, the underlying mechanism is not clear.

Impaired axonal outgrowth has been reported in SMN-depleted motor neurons [17, 29]. The low levels of SMN represented poor connection to β-actin mRNA and protein in growth cones, which took responsibility for axonal outgrowth and pathfinding defects [2, 30]. Herein we also showed a statistically significant reduction of neurite length in both SMA III and SMA I derived MNs. Meanwhile, the significant difference of neurite outgrowth between SMA III and SMA I group was observed in long-term cultures. *Ahmad* et al. suggested that the low expression of SMN activated some intracellular stress signaling pathways, like RhoA/ROCK and JNK, which may mediate neuronal growth dysfunction in SMA disease [28].

Hyperexcitability with enhanced Na^+^-channel activities in SMA mMNs has been showed, whether it is a cell autonomous event remains unknown [13, 31, 32]. Although the previous study has established a cell autonomous relationship between SMN reduction and mMNs hyperexcitability in SMA by gain- and loss-of-function study [13]. Nonetheless, *Simon* et al revealed that hyperexcitability is independent of Smn reduction in mMNs, which may relate to non-cell autonomous mechanism [32]. In the present study, hyperexcitability of neuronal activity has been observed only in SMA I derived mMNs, but not in SMA III group. Therefore, we propose that neuronal activity of MN in SMA was a comprehensive outcome from SMN deficiency resulting in cell and non-cell autonomous mechanisms.

In summary, we showed the significant difference of SMN protein, neurite outgrowth and neuronal activity between SMA I and SMA III iPSCs derived MNs. More SMA patients and age-matched contols will be enrolled to confirm these findings. In the future, the emerging genome-editing technique will be adopted to further illustrate the cause-effect relationship between SMN protein and phenotypic severity [33].

## MATERIALS AND METHODS

All studies and other procedures were approved by the Ethics Committee of the First Affiliated Hospital of Fujian Medical University (FYYY2006-01-19-01). The written informed consents were obtained from all the participants.

### Sample collection and DNA isolation

Urinary cells from the two male siblings were collected and cultured as our previous report [34]. In brief, 200-500ml of freshly midstream urine was collected in a sterile container. Urine was then centrifuged at 1000g for 5 min at room temperature, and the resulting pellets were resuspended in epithelial cell medium (EpiCM, USA). Finally, the suspension was transferred to a 25 cm^2^ cell culture flask (Corning, USA) and incubated at 37°C and 5% CO_2_ to grow robustly. The severe SMA I iPSC line was from Coriell Cell Repositories (Coriell IDs GM23240).

Genomic DNA was extracted from these cell lines using a QIAamp DNA Blood Mini Kit (Qiagen, Hilden, Germany) according to the manufacturer's instructions.

### Molecular analysis of *SMN* gene by PCR-RFLP and MLPA

PCR-RFLP was applied for screening the homozygous deletion of *SMN* gene as our previous report [35]. In brief, the exon 7/8 and flanking areas of the *SMN1* and *SMN2* genes were amplified by PCR with the primers of exon 7 (forward 5′-AGACTATCAACTTAATTTCTGATCA-3′, reverse 5′-CCTTCCTTCTTTTTGATTTTGTTT-3′) and exon 8 (forward 5′-TAATAACCAAATGCAATGTGAA-3′, reverse 5′-CTACAACACCCTTCTCACAG-3′). The PCR products were separately digested with enzyme *Dra I* and *Dde I* (Promega, USA) at 37°C overnight. The products were visualized on 3% agarose gels under UV transilluminator (Tanon, China).

MLPA was used for detection of the copy numbers of *SMN* gene [36]. Briefly, MLPA analysis was based on the commercially available SALSA MLPA kit P021 (MRC-Hollyland, Netherlands). The kit contains several probes for genes in the 5q13 region and 21 reference probes. After 4 reaction steps (denaturation, hybridization, ligation, and amplification), the products were separated on an ABI-3730 genetic analyzer (Applied Biosystems, USA) with 500 LIZ^®^ (Applied Biosystems, USA) as the internal size standard. Data were collected by Genemapper 3.0 (Applied Biosystems, California, USA) and analyzed using Coffalyser software.

### Reprogramming human urine cells into iPSC lines

Urinary cells at passage 3 were used for the reprogramming experiment. As reported previously [37], human complementary DNA sequences (cDNAs) of Oct4, Sox2, Klf4 and c-Myc were cloned into the pLV-EF1α-CDNA-IRES-EGFP vector. Lentiviruses were packaged in human embryonic kidney (HEK) 293T cells using Fugene HD transfection reagent (Roche, USA). Urinary cells were incubated in 6-well plates (2×10^5^ cells/well) and were transduced with a cocktail of lentiviruses carrying reprogramming factors plus polybrene (Sigma, USA) at an MOI=10. At 24 h post-transduction, cells were harvested by trypsinization and replated on irradiated mouse embryonic fibroblasts (MEF). On Day 2, the epithelial cell medium was replaced with the human ES medium, which was changed daily. On Day 14, culture medium was replaced with the mixed medium (human ES medium: conditional medium, at a 1:1 ratio). On Day 30, iPS colonies with a compact appearance were manually picked for further expansion and characterization. Conditional medium (CM) was collected according to previous reports [38].

### iPSCs culture and neural differentiation

All iPS cell lines were maintained in a defined xenogeneic-free culture condition [39]. Cells were cultured on Matrigel-coated plates in standard E8 medium (Thermo Scientific) and incubated at 37°C and 5% CO_2_. iPSCs were split with 0.5 mM EDTA (Cellapy) for 3 min at a ratio of 1:10 every 4-5 days.

iPSCs were differentiated into spinal motor neurons with a modified protocol [40]. iPSCs were dissociated and resuspended in differentiation medium N2B27, including DMEM/F12, Neurobasal medium at 1:1, 0.5×N2, 0.5×B27, 1×Nonessential Amino Acids and 1×Glutamax (all from Thermo Scientific). SB431542 (2μM, Torcris), LDN 193189 (0.3 μM, Stemgent) and CHIR99021 (3μM, Stemgent) were added to the medium for 4 days. The culture medium was changed every other day. On Day 4, the EBs were treated with retinoic acid (RA, 0.1 μM, Sigma) and smoothened agonist (SAG, 0.5 μM, Millipore) for MN induction. On Day 9, the neurospheres were dissociated into single cell with Accutase (Thermo Scientific) and plated on poly-ornitine and laminin (Sigma) double-coated coverslips with the same medium in the presence of DAPT (10μM, Abcam) for 72 h to generate pMNs. Neurotrophic factors (BDNF, GDNF, cAMP, at 10 ng/ml; IGF-1, at 1μM; AA, at 200 ng/ml) were added to the differentiation medium on Day 12 to Day31. The culture medium was changed every two days.

In addition, for the generation of postmitotic γ-aminobutyric acid (GABA) neurons, cyclopamine (0.5μM, Abcam) was added in place of SAG on Day4.

### Alkaline phosphatase staining, immunocytochemistry and quantification

For detection of alkaline phosphatase (AP) activity, the AP Detection Kit (Millipore) was used according to the manufacturer's recommendations with positive staining showing pink or purple for AP.

The cultures of iPSCs and MNs were fixed with 4% paraformaldehyde (10-20 min at room temperature), rinsed three times with 1× PBS (Medicago AB) and incubated in a blocking buffer (10% donkey serum and 0.2% triton X-100 in PBS) for 1hour and then incubated with primary antibodies (Supplementary Table 2) overnight in a refrigerator at 4°C. Fluorescence conjugated secondary antibodies (Thermo Scientific) and DAPI (Sigma) were used at 1:1,000 dilution. Images were captured by a Leica TCS SP8 confocal system.

Image-J software (NIH, MD, USA) was used for further quantification of the cell population. Cell counting was performed by a person blind to the experiment and replicated in five random visual fields from three independent experiments.

### Neurite length measurement

The neurospheres were dissociated into small clones, and were plated on poly-ornitine and laminin double-coated coverslips. The specific neurite of motor neuron was identified using anti-Tau and HB9 (1:100) co-immunostaining on Day 2, Day 3, Day 5 and Day 7 after attachment by a Leica TCS SP8 confocal system. The total length of neurite and average neurite outgrowth rate were analyzed by a blinded observer using ImageJ-NeuronJ-Tracing software. Neurite total length was measured from the edge of the cluster to the visually distinguishable end point. The calculated interval of neutite outgrowth rate was divided into two segments: the previous three days after attachment (Day 0 to Day 3) and the last four days after attachment (Day 3 to Day 7). At least 50 cells were calculated in each group.

### Electrophysiology

Whole-cell patch-clamp recordings were performed on iPSC-derived CHAT^+^ neurons on Day 31 after iPSC differentiation as our previous report [41]. Coverslips were placed in a bath solution made of the following (in mM): NaCl 135, KCl 3, CaCl_2_ 2, MgCl_2_ 1, glucose1 1, sucrose 10 and HEPES10, pH 7.4 adjusted with NaOH at 310mOsm. Tetrodotoxin (TTX, 0.5μM) was attained using a gravity-fed drug barrel system and was diluted in extracellular solution. The patch pipettes (3–5MΩ) contained (in mM): KCl 140, NaCl 9, MgCl_2_ 1, EGTA 0.2, ATP-Mg 2, GTP-Na 0.25, and HEPES10, pH 7.2 adjusted with KOH at 290mOsm. All chemicals were purchased from Sigma-Aldrich. Neurons were visualized using a Nikon ECLIPSETi microscope (Tokyo, Japan) with differential interference contrast optics at 40X. Voltage-clamp and current-clamp recordings were obtained using an Axon Multiclamp 700B Patch Clamp Amplifier (Molecular Devices, Sunnyvale, CA). Signals were filtered at 1 kHz and sampled at 10 kHz using a Digidata1440A analog-to-digital converter (Molecular Devices). All data were saved on a computer hard disk and analyzed with pClamp10.2 (Molecular Devices). Capacitance and series resistance were compensated (typically 30%). All recordings were conducted at RT (22°C–24°C).

### Western blot analysis

Cells were harvested in lysis buffer (Sigma) supplemented with a 1% protease inhibitor PMSF (Beyotime) and centrifuged at 13,000 rpm for 20 minutes at 4°C. Protein extracts were separated on 12% SDS-PAGE gels and immunoblotted with primary antibodies SMN (1:500, Santa Cruz) and Tubulin (1:10,000, Beyotime). HRP-conjugated secondary antibodies (1:5,000, Santa Cruz) were used to detect primary antibodies and proteins were visualized by chemiluminescence (Millipore).

### RNA isolation and quantitative real-time polymerase chain reaction

Total RNA from iPSCs and EBs were extracted using a Trizol kit (Invitrogen) according to the manufacturer's instructions. A 0.5-μg aliquot of total RNA from each sample was reversely transcribed into cDNA. Quantitative PCRs were performed with SYBR^@^*Premix EX Taq*^TM^ II kit (Takara) and run on an Applied Biosystems Step one Real-time PCR System. Primers for real-time PCR were listed in Supplementary Table 3.

### Teratoma assays

iPSC clones were manually detached by collagenase IV (Thermo Scientific, USA) treatment and injected intramuscularly into 8- to 10-week-old non-obese diabetic/severe combined immune deficient (NOD/SCID) mice (approximately 3×10^6^ cells per site). Teratomas were harvested approximately 4 months later and processed for hematoxylin-eosin staining.

### Karyotyping

High-resolution Giemsa-banding from each iPS cell line was assessed for chromosomal rearrangements, using standard protocol.

### Embryoid body (EB) formations assay

iPSCs were harvested by EDTA treatment and grown in suspension to obtain EBs for eight days. The cells were further plated in Matrigel-coated 6 cm dishes (Corning) and cultured for another eight days before collection.

### DNA fingerprinting analysis

To confirm that these iPSCs were derived from the subject's urine cells, short tandem repeat (STR) analysis (see Supplementary Table 1) was performed with the Golden*eye*^TM^20A STR kit (Dingsheng High Technology, Inc.) and analyzed on an ABI3100 genetic analyzer.

### Statistical analysis

All data were obtained from at least three independent experiments, unless otherwise indicated. SPSS 16.0 was used for statistical comparisons. All data are presented as the mean± SEM, and significance was determined using the one-way ANOVA with LSD *post hoc* test. Differences were considered statistically significant when the P value was less than 0.05 (*), 0.01 (**) or 0.001 (***).

## SUPPLEMENTARY FIGURES AND TABLES


