# Supplementary material for: Modeling the differential phenotypes of spinal muscular atrophy with high-yield generation of motor neurons from human induced pluripotent stem cells
Source: Oncotarget. 2017 Jan 31;8(26):42030–42. doi: 10.18632/oncotarget.14925 (PMC5522047; doi:10.18632/oncotarget.14925)
Supplement: Supplementary file 1 [file oncotarget-08-42030-s001.pdf]

Modeling the differential phenotypes of spinal muscular atrophy with high-yield generation of motor neurons from human induced pluripotent stem cells

SUPPLEMENTARY MATERIALS

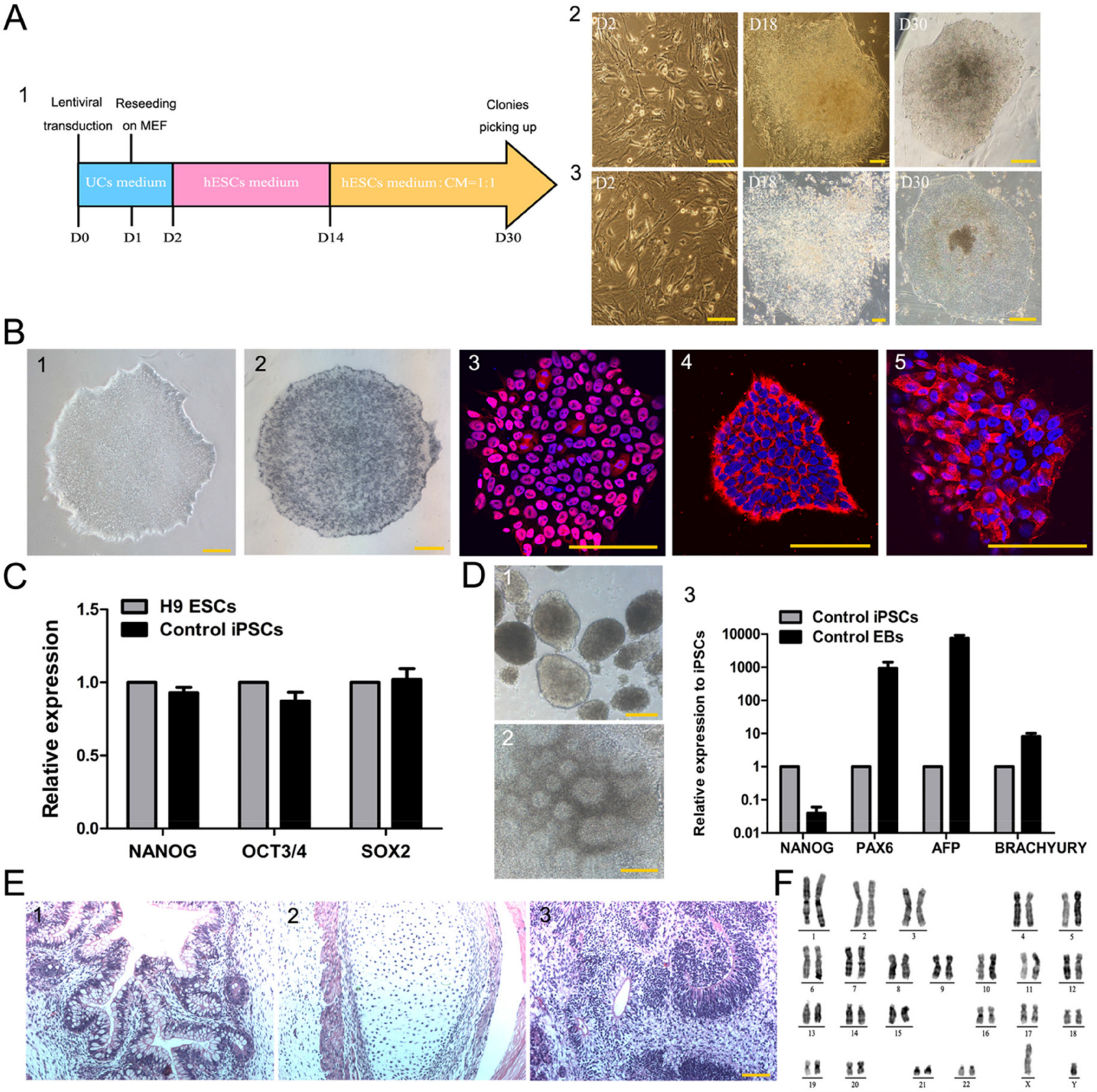

Supplementary Figure 1: Generation and characterization of iPSCs derived from urine cells of the two male siblings. **A**. Schematic representation of the iPSCs generation protocol (1). Sequential morphological changes of reprogrammed urine cells on day 2, 18 and 30 (2, 3). **B.** AP staining and immunofluorescence of Control iPSCs (1) show the expression of the pluripotent markers: AP (2), NANOG (3), SSEA4 (4) and TRA-1-60 (5). **C.** The endogenous expression of the pluripotency genes (NANOG, OCT3/4 and SOX2) was quantified by qRT-PCR. Gene expression was normalized to the H9 ESCs, which was arbitrarily set to 1. **D.** Phase contrast photographs show the formation of EBs (1) at day 8 and rosettes (2) at day 16 from iPSCs, and qPCR detected the genes PAX6 (ectoderm), AFP (endoderm) and BRACHYURY (mesoderm) reflecting the 3-germ layer differentiation of EBs (3). **E.** The sections of teratomas were stained with hematoxylin-eosin: endoderm (1), mesoderm (2) and ectoderm (3). **F.** Representative karyotypes of Control iPS colonies. Data presented as the mean ± SEM. n=3. Scale bar, 100 μm.

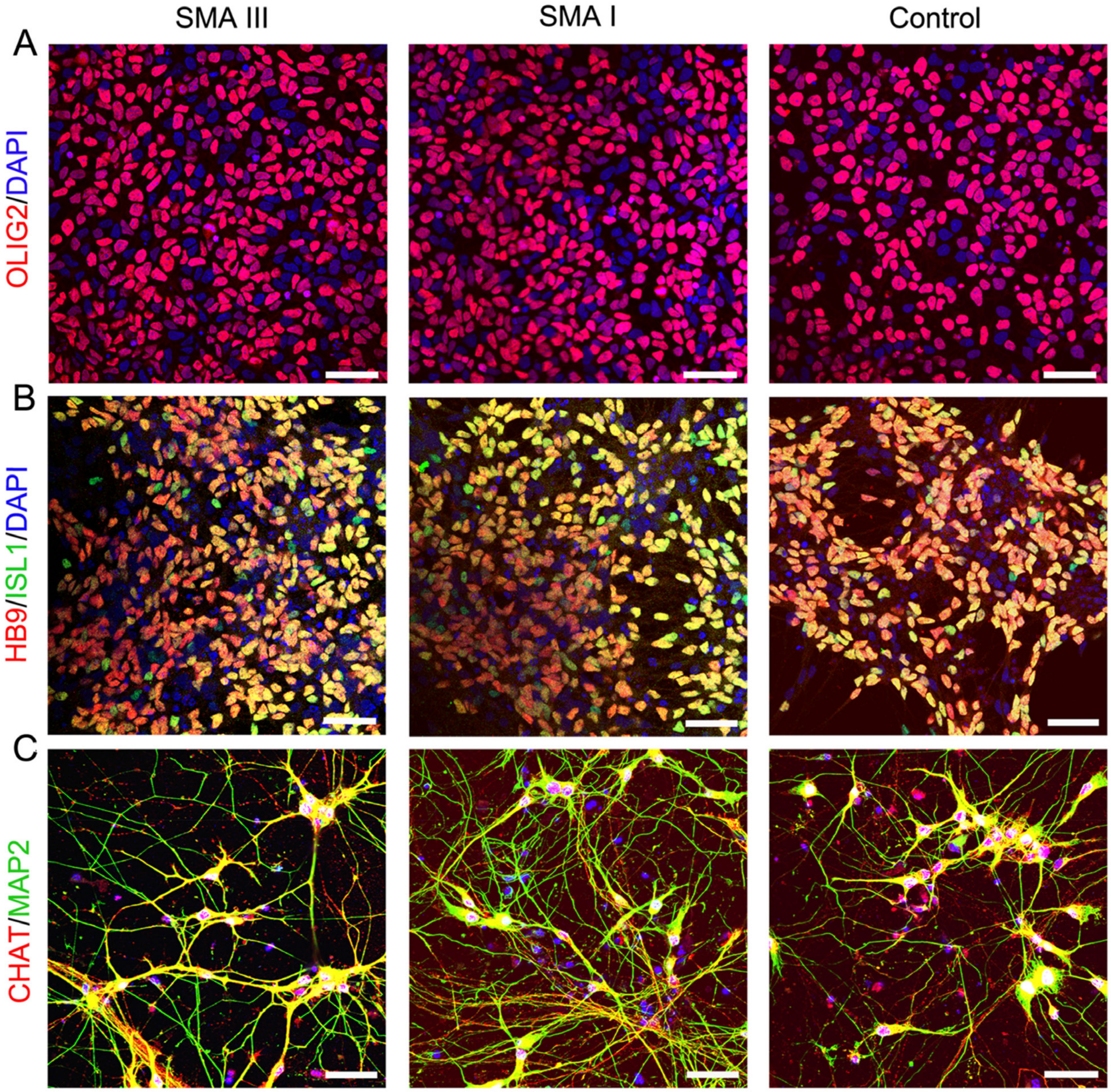

Supplementary Figure 2: SMA III and SMA I iPSCs generate MNs as efficiently as control iPSCs. A. Representative images of MNPs, identified by OLIG2 at day 10 after differentiation. **B.** Representative postmitotic MNs, identified by HB9 and ISL1 at day 12 after differentiation. **C.** Representative mature MNs, identified by CHAT and MAP2 at day 31 after differentiation. Scale bar, 50 µm.

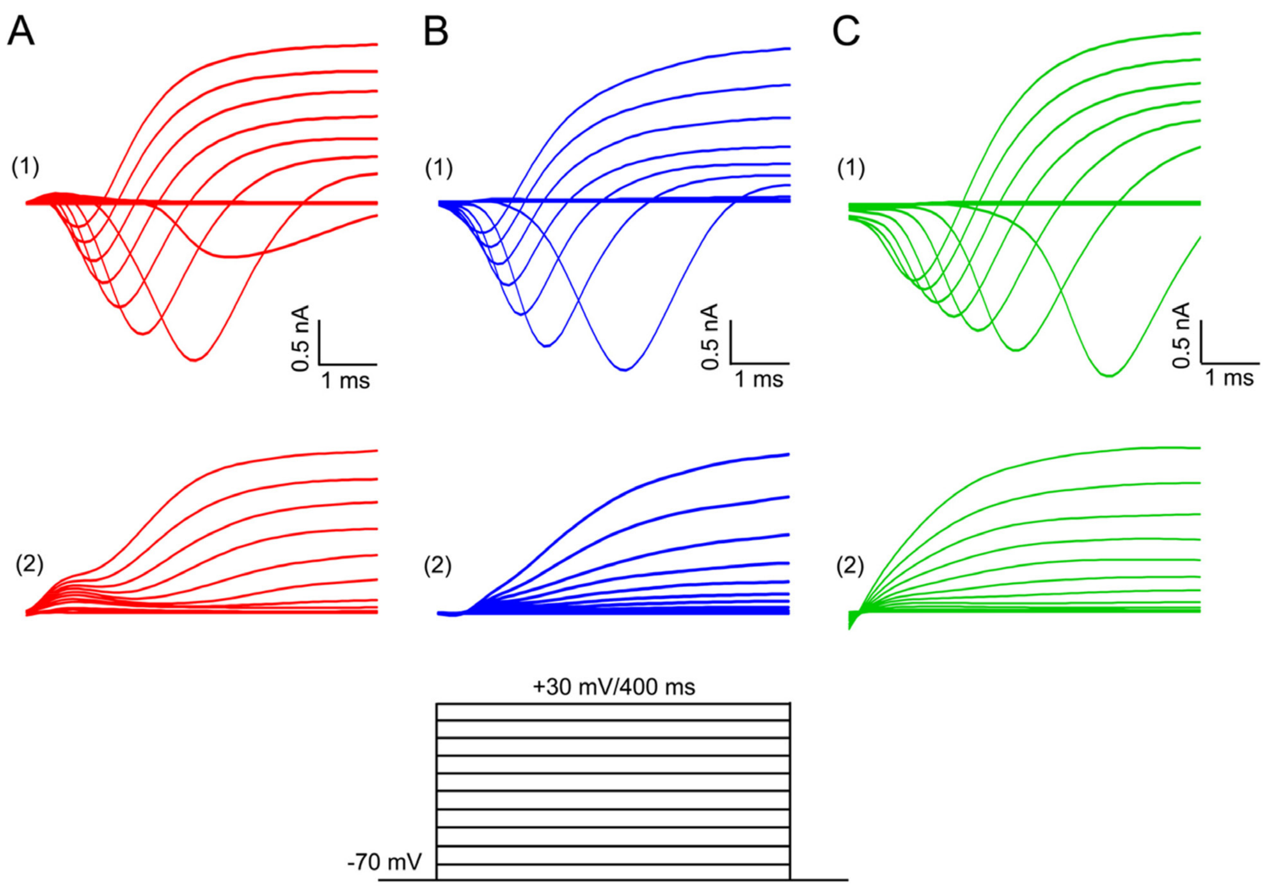

Supplementary Figure 3: Motoneurons differentiated from SMA III, SMA I and control-iPSCs gain electrophysiological maturity. A-C. 1. Large inward Na^+ currents (I_{Na}) and outward K^+ currents (I_k) were elicited with a holding potential of -70 mV by 10 mV steps to potential of +30 mV in 400 ms duration for SMA III (red, A.1), SMA I (green, C.1) and Control (blue, B.1) derived MNs. A-C, 2. Representative traces of I_{Na} were effectively blocked by Tetrodotoxin (TTX, 0.5 μM) for each group.

Supplementary Table 1: STR analysis of iPSCs derived from the two male siblings' urine cells

STR Locus	STR repetitions			
	SMA urine cells	SMA iPSC	Control urine cells	Control iPSC
D5S818	11,12	11,12	10,11	10,11
D21S11	30,32	30,32	30,32.2	30,32.2
D18S51	12,13	12,13	13,19	13,19
D3S1358	16,17	16,17	15,16	15,16
D13S317	9,11	9,11	8,11	8,11
D7S820	8,11	8,11	10,11	10,11
D16S539	9,12	9,12	9,12	9,12
CSF1PO	11,12	11,12	12,12	12,12
Penta_D	9,11	9,11	9,11	9,11
vWA	14,16	14,16	14,16	14,16
D8S1179	10,10	10,10	10,14	10,14
TPOX	8,8	8,8	8,8	8,8
Penta_E	5,14	5,14	12,20	12,20
TH01	8,10	8,10	6,10	6,10
FGA	20,26	20,26	22,26	22,26
Amelogenin	X,Y	X,Y	X,Y	X,Y

Supplementary Table 2: Primary antibodies related to immunocytochemistry

Antibody	Isotype	Dilution	Source
NANOG	Goat IgG	1:1000	R&D
SSEA4	Mouse IgG	1:400	DSHB
TRA-1-60	Mouse IgG	1:1000	Millipore
PAX6	Rabbit IgG	1:400	Covance
SOX2	Goat IgG	1:1000	R&D
OLIG2	Rabbit IgG	1:300	Abcam
TUJ1	Mouse IgG	1:10000	Covance
HB9	Goat IgG	1:50	Santa Cruz
ISLET1	Mouse IgG	1:100	DSHB
MAP2	Rabbit IgG	1:10000	Santa Cruz
CHAT	Goat IgG	1:300	Millipore
SYNAPTOPHYSIN	Mouse IgG	1:2000	Millipore
GABA	Rabbit IgG	1:2000	Sigma
Anti-Tau	Rabbit IgG	1:500	Abcam

Supplementary Table 3: Primer sequences for real-time PCR. All the real-time PCR used primers were tested with standard curve, amplification efficiency was between 95%–105%, and the R^2 for linear relationship is >0.999

Gene	Forward primer sequences (5'→3')	Reverse primer sequences (5'→3')
NANOG	TGAACCTCAGCTACAAACAG	TGGTGGTAGGAAGAGTAAAG
OCT3/4	CCTCACTTCACTGCACTGTA	CAGGTTTTCTTTCCCTAGCT
SOX2	CCCAGCAGACTTCACATGT	CCTCCCATTTCCCTCGTTTT
PAX6	TTGCTTGGGAAATCCGAG	TGCCCGTTCAACATCCTT
AFP	ATTGGCAAAGCGAAGCTG	GCTGTGGCTGCCATTTTT
BRACHYURY	CCCTATGCTCATCGGAACA	TTCCAAGGCTGGACCAAT
SMN-FL	ATGTTAATTTCATGGTACATG	GGAATGTGAGCACCTTCCTTC
GAPDH	ATGACATCAAGAAGGTGGTG	CATACCAGGAAATGAGCTTG
